# Supplementary figures and images for: Identification and mechanistic insights of cell senescence-related genes in psoriasis
Source: PeerJ. 2025 Jan 14;13:e18818. doi: 10.7717/peerj.18818 (PMC11740738; doi:10.7717/peerj.18818)

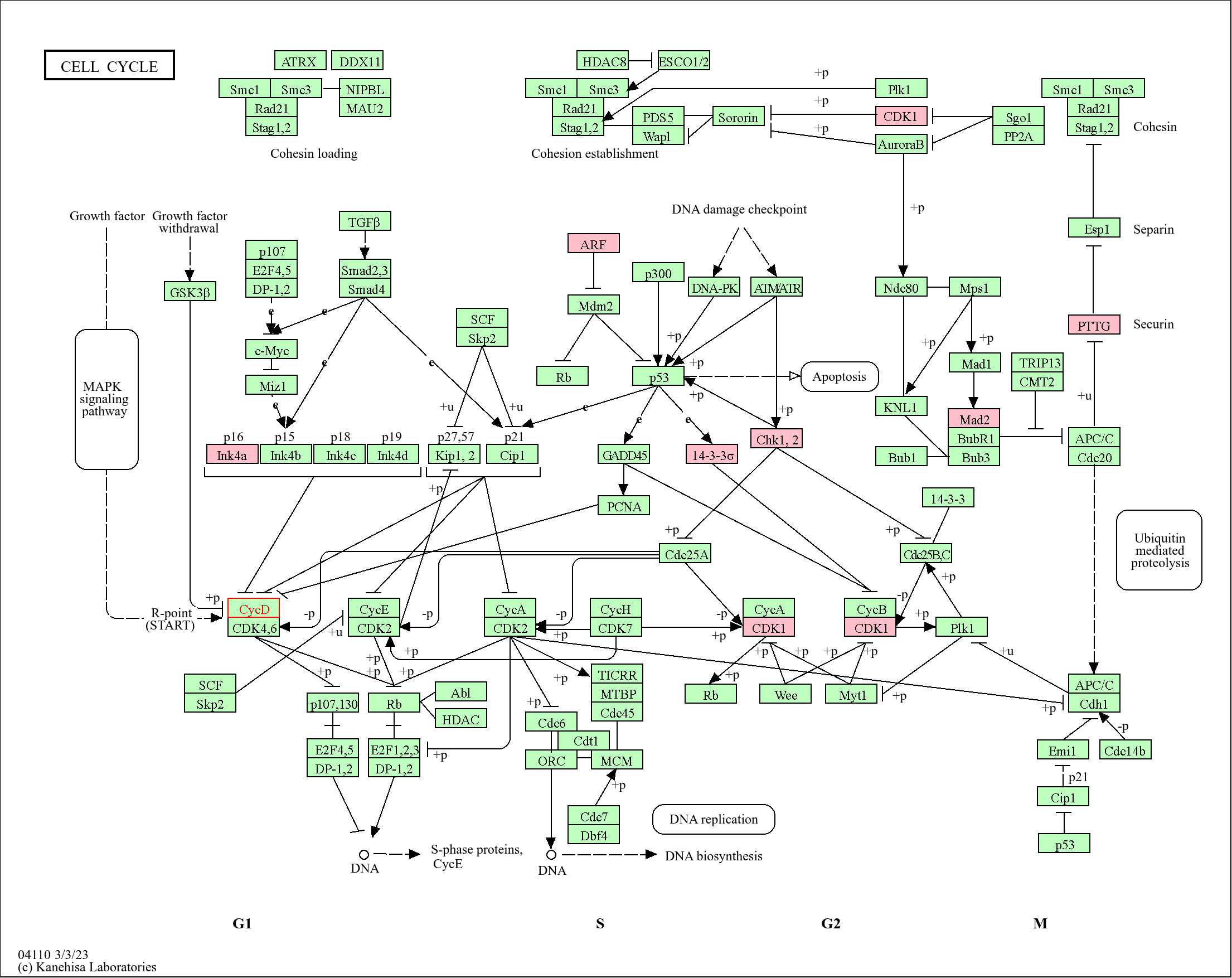

Supplement: Supplemental Information 3 [file peerj-13-18818-s003.zip › supplementary material 2/hsa04110@2x_20240724_152130.png]

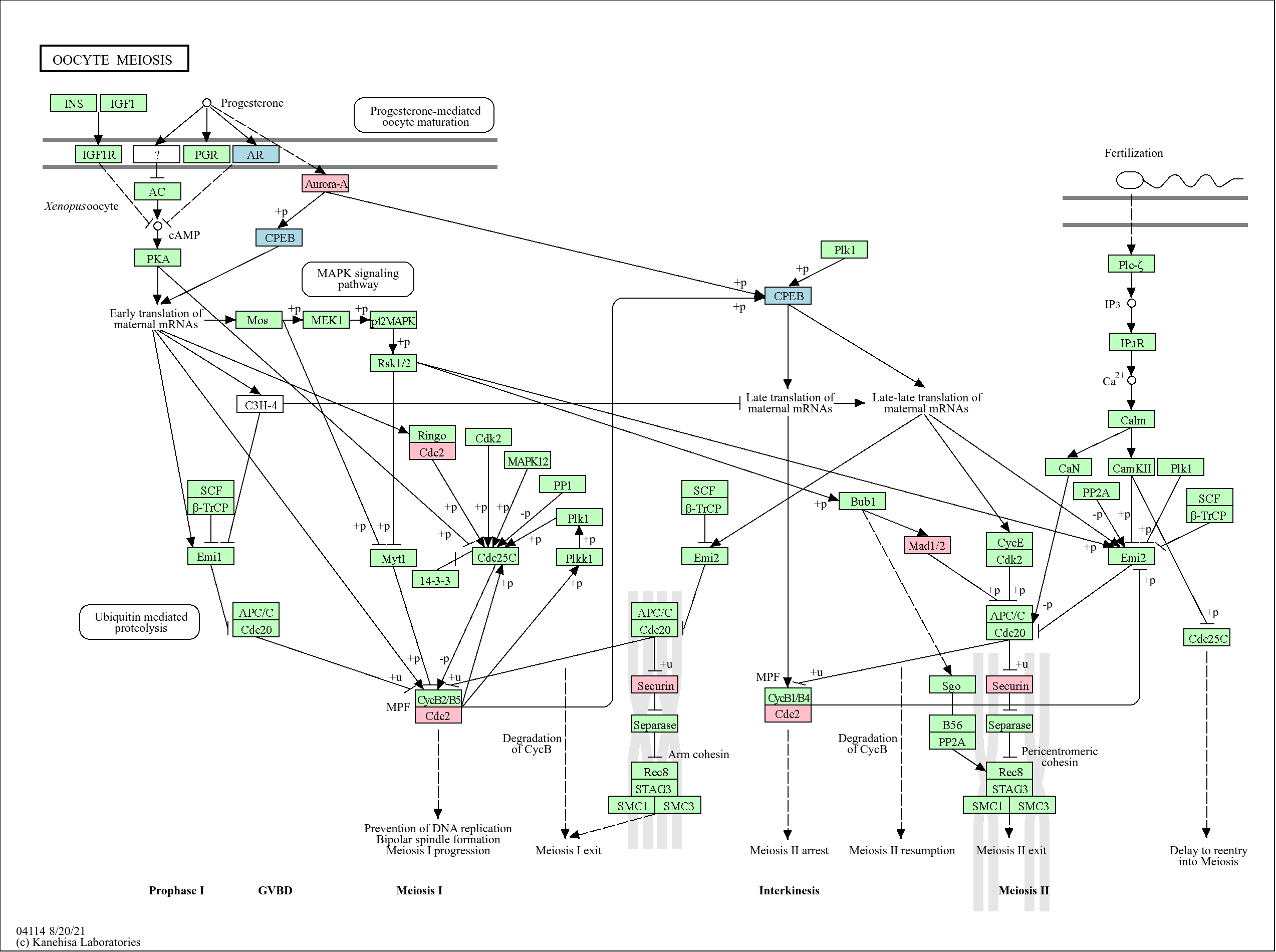

Supplement: Supplemental Information 3 [file peerj-13-18818-s003.zip › supplementary material 2/hsa04114@2x_20240724_152809.png]

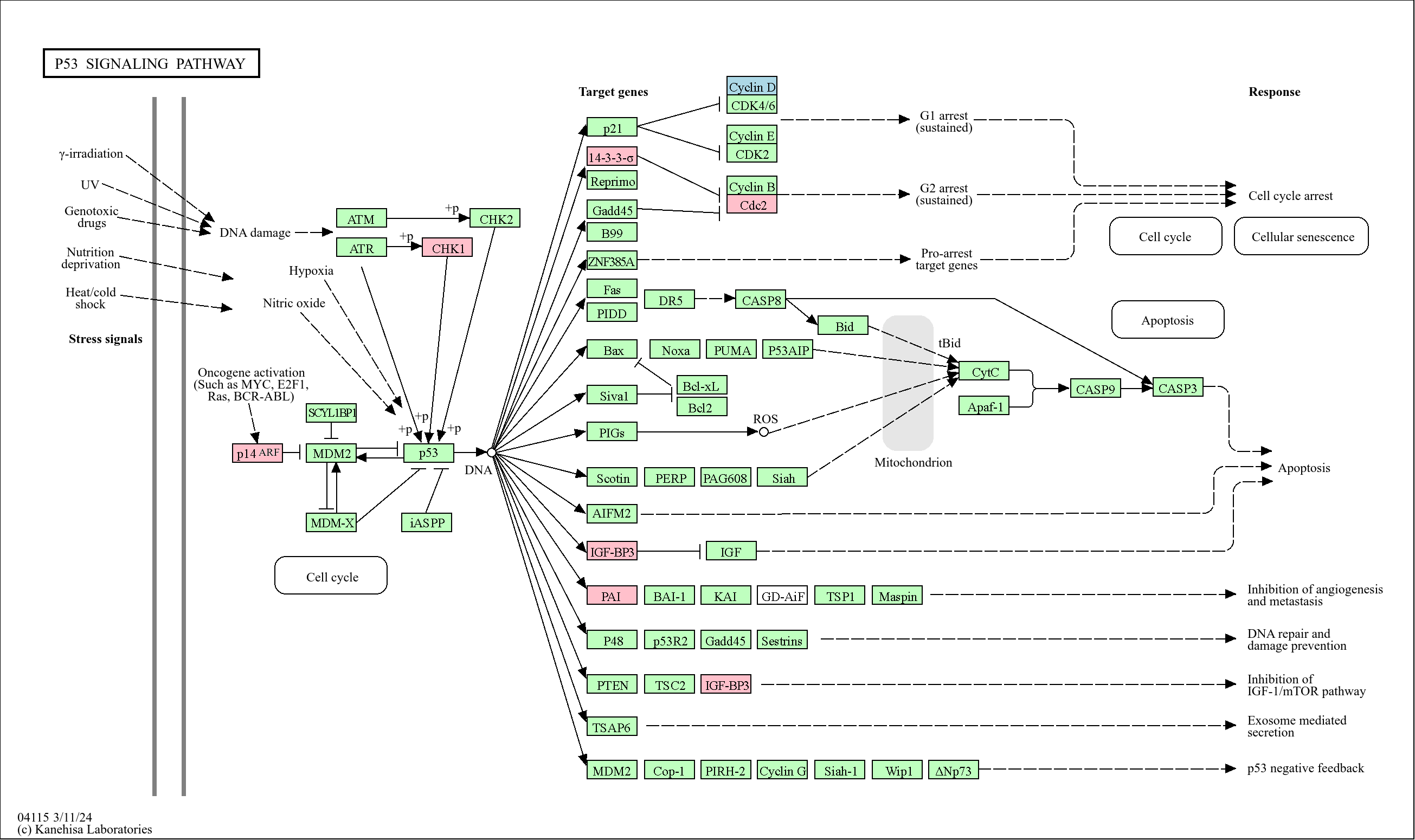

Supplement: Supplemental Information 3 [file peerj-13-18818-s003.zip › supplementary material 2/hsa04115@2x_20240724_152854.png]

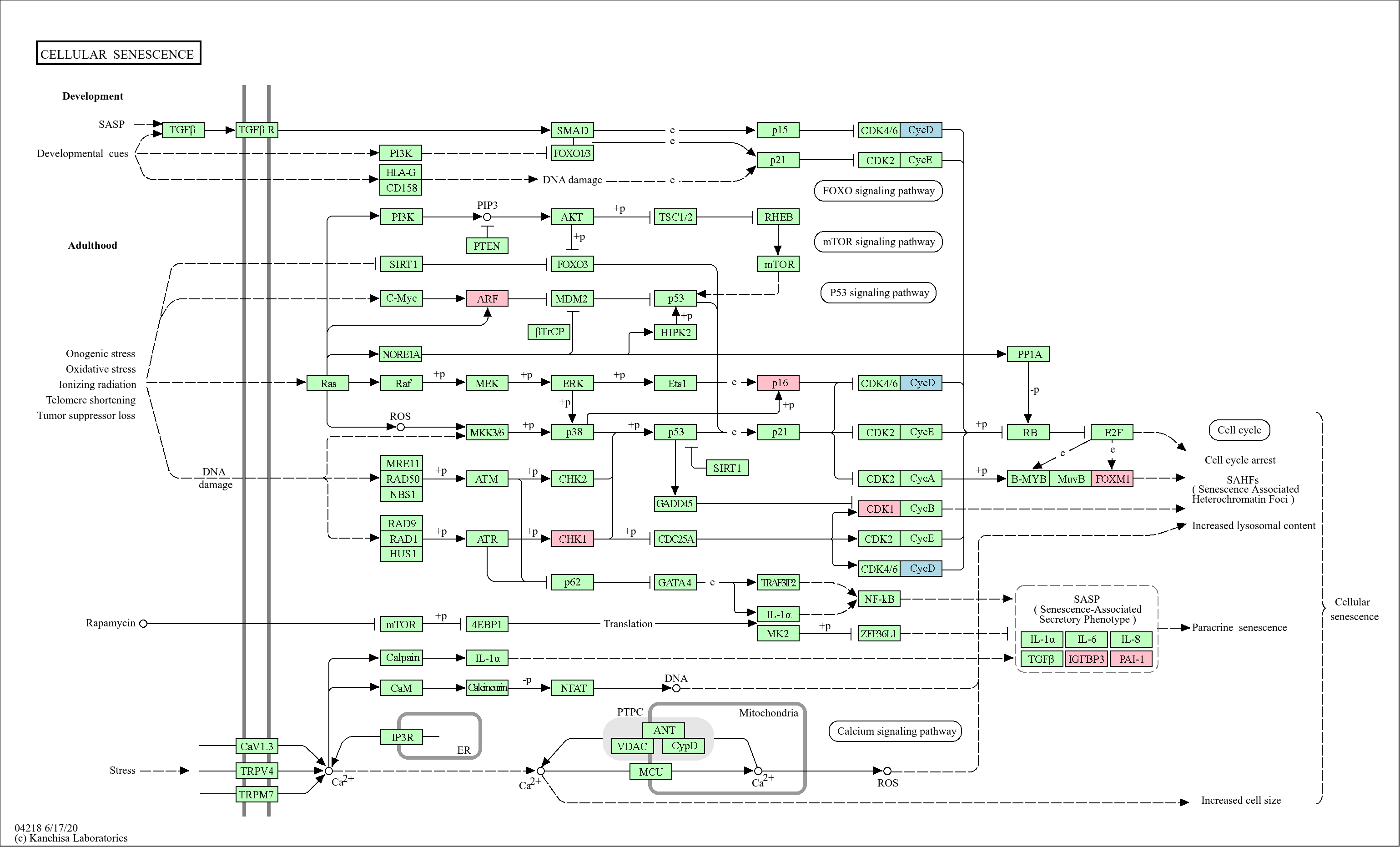

Supplement: Supplemental Information 3 [file peerj-13-18818-s003.zip › supplementary material 2/hsa04218@2x_20240724_152943.png]

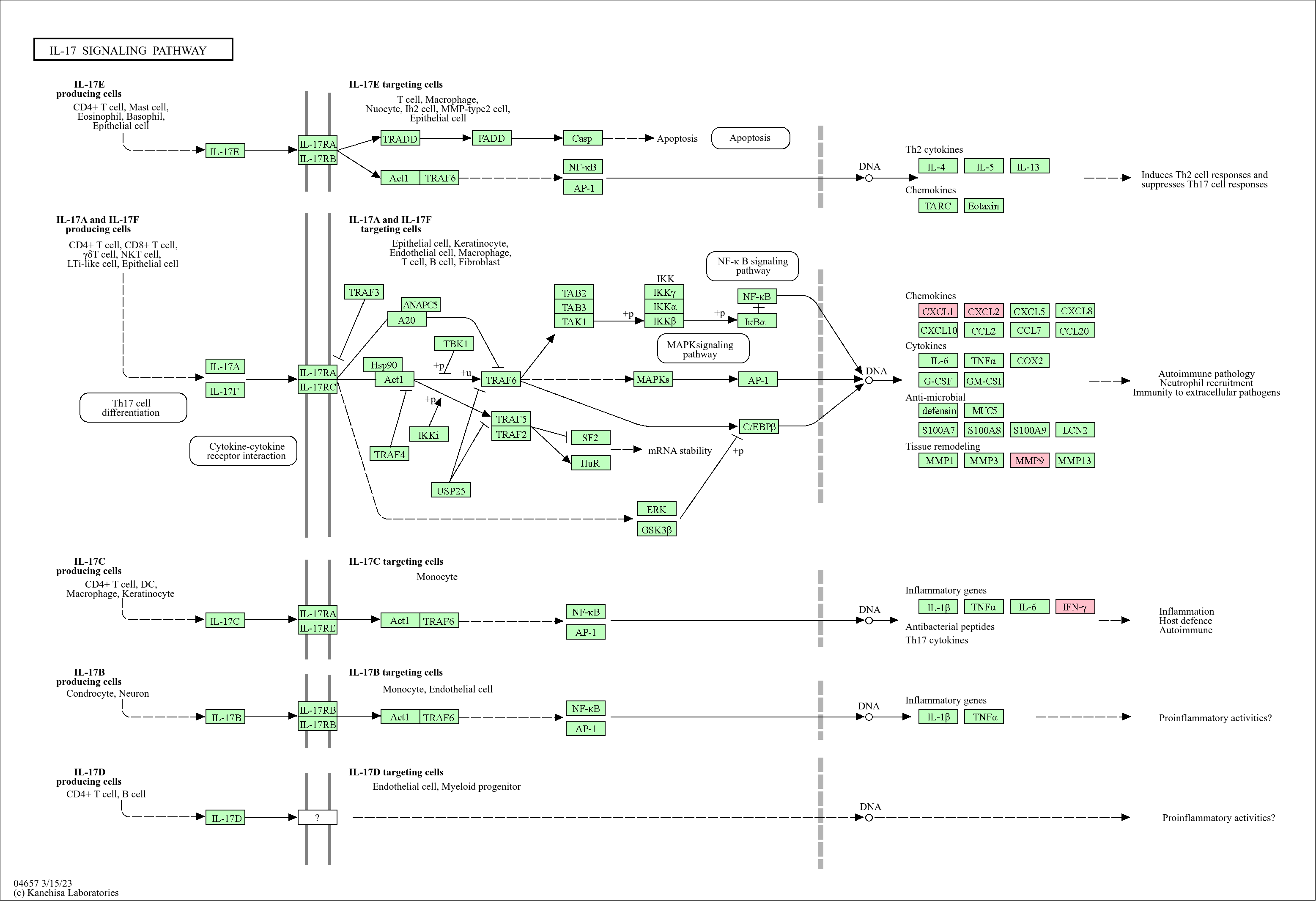

Supplement: Supplemental Information 3 [file peerj-13-18818-s003.zip › supplementary material 2/hsa04657@2x_20240724_153035.png]

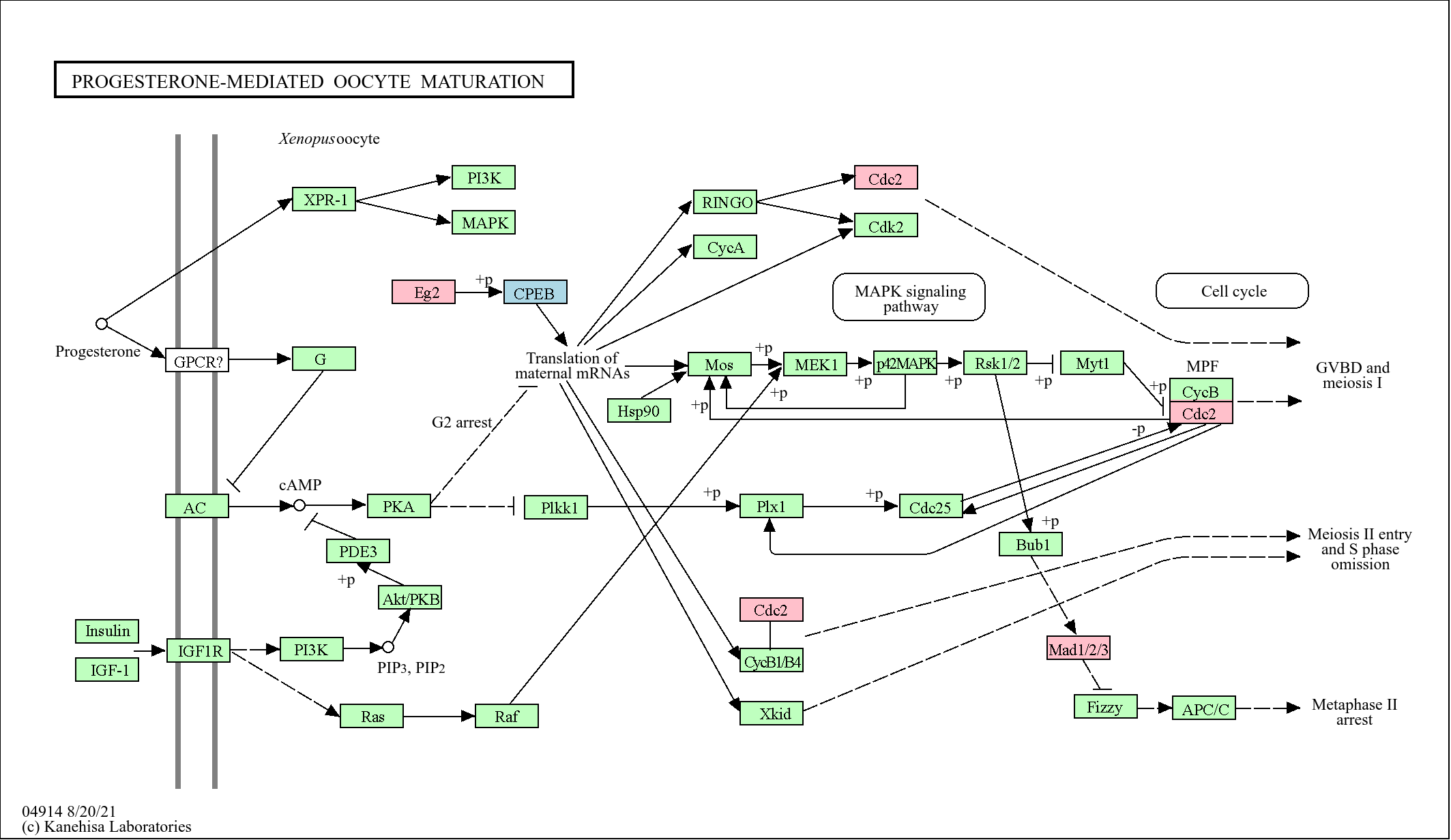

Supplement: Supplemental Information 3 [file peerj-13-18818-s003.zip › supplementary material 2/hsa04914@2x_20240724_153106.png]

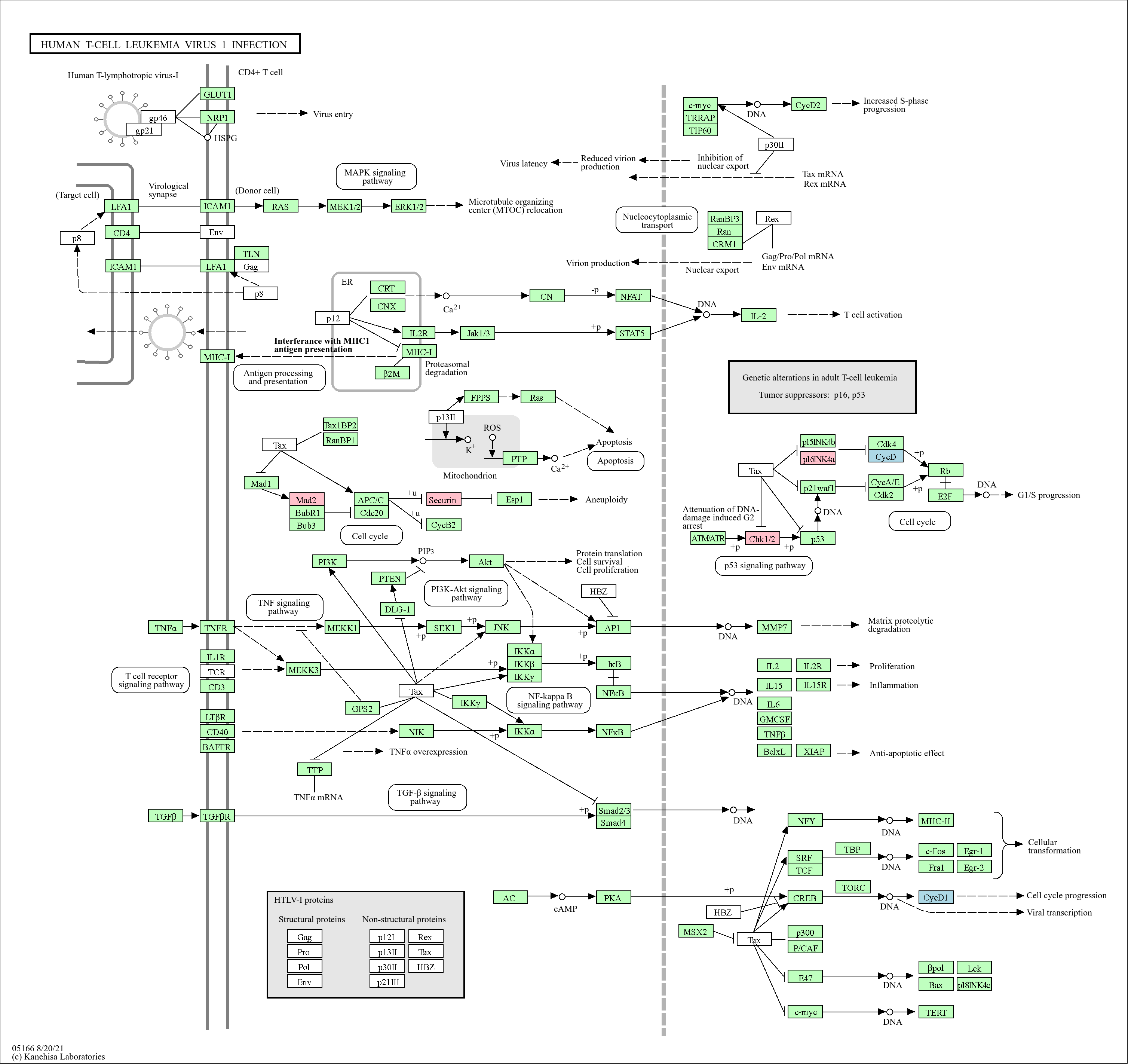

Supplement: Supplemental Information 3 [file peerj-13-18818-s003.zip › supplementary material 2/hsa05166@2x_20240724_153144.png]

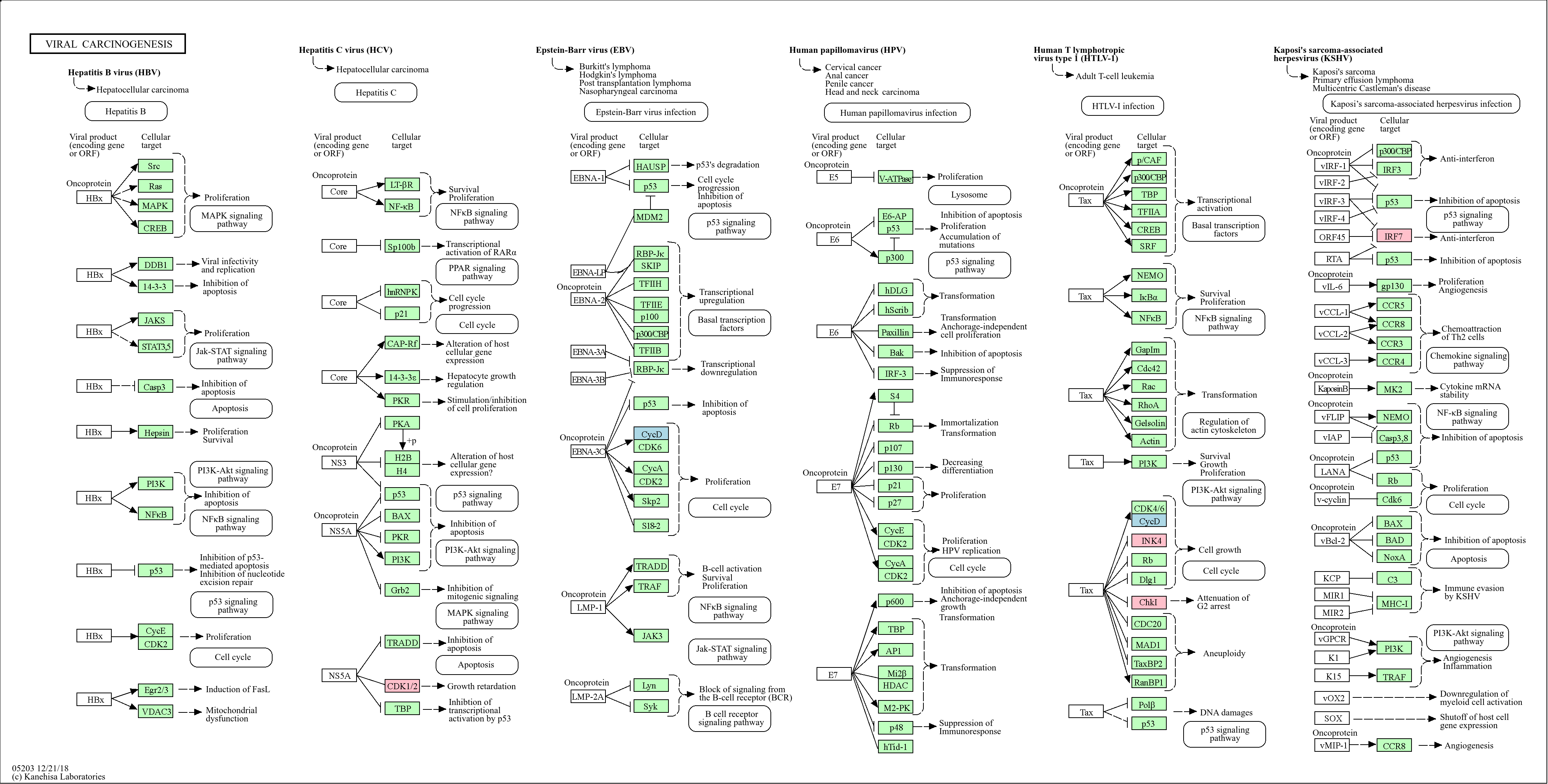

Supplement: Supplemental Information 3 [file peerj-13-18818-s003.zip › supplementary material 2/hsa05203@2x_20240724_153220.png]

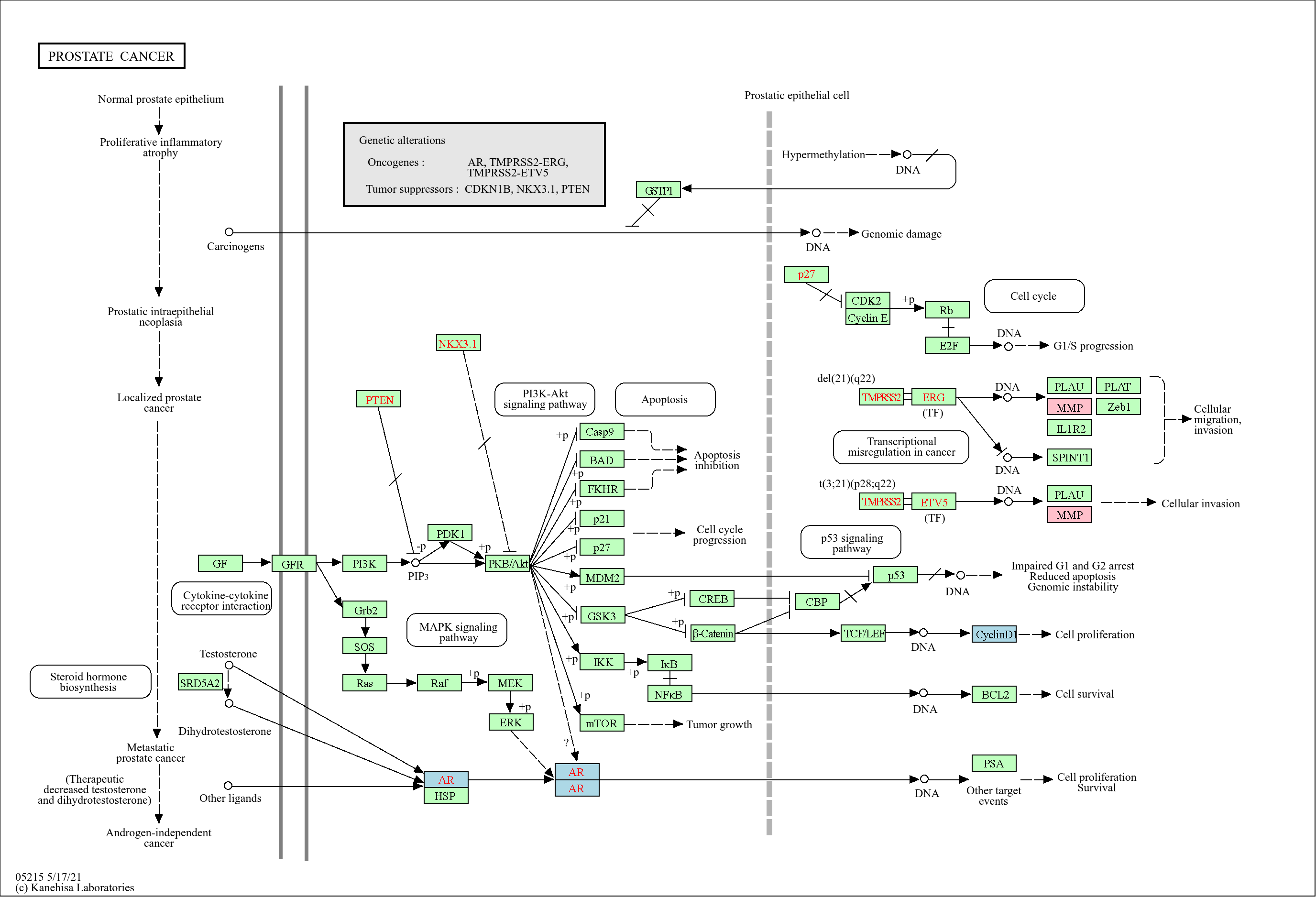

Supplement: Supplemental Information 3 [file peerj-13-18818-s003.zip › supplementary material 2/hsa05215@2x_20240724_153308.png]

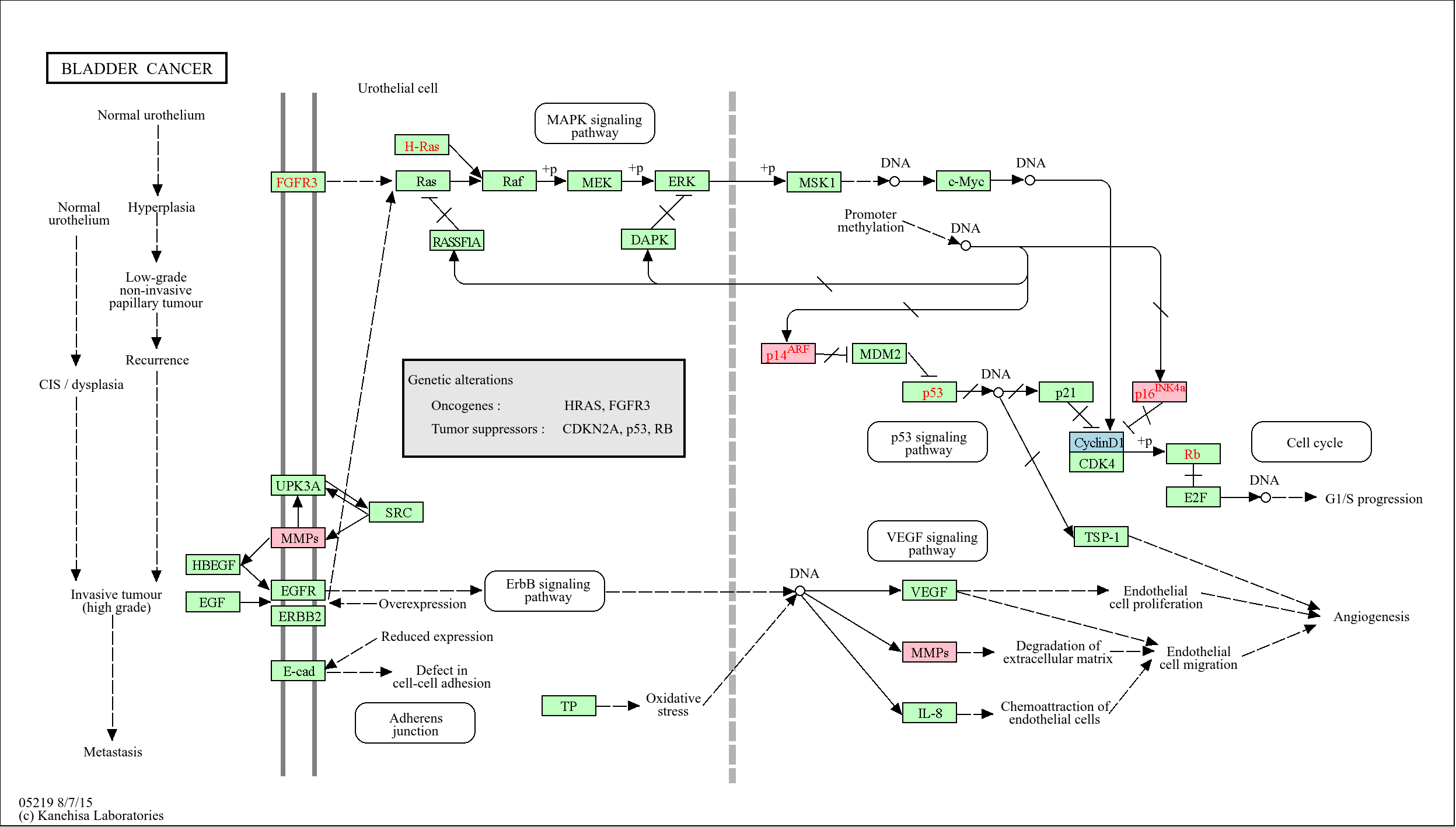

Supplement: Supplemental Information 3 [file peerj-13-18818-s003.zip › supplementary material 2/hsa05219@2x_20240724_153333.png]
